# Research on Improved Algorithms for Cone Bucket Detection in Formula Unmanned Competition

**DOI:** 10.3390/s24185945

**Published:** 2024-09-13

**Authors:** Xu Li, Gang Li, Zhe Zhang, Haosen Sun

**Affiliations:** School of Automobile and Traffic Engineering, Liaoning University of Technology, Jinzhou 121001, China; 221291002@stu.lnut.edu.cn (X.L.); zechina@163.com (Z.Z.); shs1234562024@163.com (H.S.)

**Keywords:** target detection, deep learning, model lightweight, multi-stage knowledge distillation network

## Abstract

The model network based on YOLOv8 for detecting race cones and buckets in the Formula Unmanned Competition for Chinese university students needs help with problems with complex structure, redundant number of parameters, and computation, significantly affecting detection efficiency. A lightweight detection model based on YOLOv8 is proposed to address these problems. The model includes improving the backbone network, neck network, and detection head, as well as introducing knowledge distillation and other techniques to construct a lightweight model. The specific improvements are as follows: firstly, the backbone network for extracting features is improved by introducing the ADown module in YOLOv9 to replace the convolution module used for downsampling in the YOLOv8 network, and secondly, the FasterBlock in FasterNet network was introduced to replace the fusion module in YOLOv8 C2f, and then the self-developed lightweight detection head was introduced to improve the detection performance while achieving lightweight. Finally, the detection performance was further improved by knowledge distillation. The experimental results on the public dataset FSACOCO show that the improved model’s accuracy, recall, and average precision are 92.7%, 84.6%, and 91%, respectively. Compared with the original YOLOv8n detection model, the recall and average precision increase by 2.7 and 1.2 percentage points, the memory is half the original, and the model computation is 51%. The model significantly reduces the misdetection and leakage of conical buckets in real-vehicle tests and, at the same time, ensures the detection speed to satisfy the deployment requirements on tiny devices. Satisfies all the requirements for deployment of tiny devices in the race car of the China University Student Driverless Formula Competition. The improved method in this paper can be applied to conebucket detection in complex scenarios, and the improved idea can be carried over to the detection of other small targets.

## 1. Introduction

In the Chinese University Student Driverless Formula Competition, the car needs to perceive the surrounding environment to achieve subsequent planning and control, and the color of the cone bucket in the competition is mainly red, yellow, and blue [1]. How to adopt an effective and efficient algorithm is the key to winning.

The first-stage detection algorithm is the first choice for target detection due to its detection speed and detection efficiency, while the first-stage detection algorithm is mainly YOLO; YOLOV5 [2] will be launched in 2020 by introducing the CSP (Cross Stage Partial networks) module. The detection accuracy and inference speed are significantly improved. In addition, Mosaic data enhancement and adaptive anchor frame calculation are used to enhance the model’s ability to detect objects of different sizes and angles. However, it still needs to improve its performance when dealing with objects of extreme proportions. The detection accuracy of small objects is limited, and the generalization ability in complex scenes is relatively weak.

YOLOv7 further improves detection accuracy by using a new network architecture and loss function. Especially in the area of small object detection, but the complexity of its version leads to a high demand for training resources, especially more training time and computing resources, which limits its application in rapid development and deployment.

YOLOv8 algorithm introduces an Anchor-Free detection method, simplifies the model architecture, and improves the model’s detection ability of small objects. Secondly, YOLOv8 significantly improves the flexibility and efficiency of the model through the enhancement of feature extraction. Although YOLOv8 has achieved technical breakthroughs in many aspects, there is still room for improvement in detection accuracy in extremely dense scenarios.

YOLOv9 further optimizes the real-time performance and reduces the computational overhead, but in some complex scenes, especially when multiple small objects stick, its separation ability is insufficient.

YOLOv10 introduces innovative techniques such as NMS-free (non-maximum suppression) training and large kernel convolution, significantly reducing inference time while maintaining high accuracy. In addition, its lightweight classification head and spatial channel decoupling downsampling further improve the overall efficiency of the model. Although YOLOv10 has excellent performance in many aspects, due to its adoption of many novel technologies, there are high technical requirements for users, increasing the difficulty of model understanding and application. Therefore, this paper chooses the YOLOv8 algorithm with the most widely applied effect as the baseline model.

Since the original YOLOv8 algorithm can not detect cone buckets well, this paper improved the algorithm to make it meet the requirements of deploying small devices with high detection accuracy and less false detection and leakage. In terms of model improvement, Zhuo Jiayue et al. [3] improved cone bucket detection based on YOLOv5. Firstly, CA attention was introduced, then the color space transformation module was modified, and finally, the Gaussian Wasserstein loss function was adopted, thus improving the accuracy of the improved model by 6.9% and the recall rate by 4.4%. The average accuracy is increased by 4.9%. These improvements not only improve the detection accuracy of cone buckets but also strengthen the detection ability of small targets and color difference recognition ability, making the model more reliable and efficient in practical application. Wang Gang [4] et al. improved detection in UAV scenarios on the basis of YOLOv8. Firstly, IOU was improved, and Wise-IoU was introduced as a loss function. Secondly, BiFormer attention was added to optimize the backbone network. Finally, in order to reduce the missing rate of small targets, the pyramid of feature extraction was modified, and the Focal FasterNet module modified on FasterNet was introduced so that the average accuracy of the improved model was increased by 7.7%. These improvements made the algorithm more suitable for the detection of small targets under UAVs. Li Ping et al. [5] improved tomato maturity detection on the basis of YOLOv8 and adopted the MHSA attention mechanism to improve the YOLOv8 network. Thus, the Precision, Recall, and mAP50 of the improved model are improved from the original 0.806, 0.807, and 0.864 to 0.990, 0.960 and 0.916, respectively. These improved measures make the model realize the efficient fusion of semantic information and detail information and enhance the ability of the network to capture the key features of the target so that the improved model is more suitable for the detection of tomato maturity.

However, deep learning models usually account for the memory is often very large, it is challenging to deploy in mobile devices embedded devices; in Chinese college students, unmanned formula competition is usually used in small computers, and due to the unmanned direction of the environment perception is only the most first step, followed by path planning, tracking and control need to run algorithms, so the need for environment perception algorithms can not be too much occupied running memory! Therefore, lightweight research on the model is becoming more and more critical. Liu Yulin [6] and others improve the YOLOP algorithm for lane line detection; firstly, the lightweight backbone network EfficientNet-V2 is used to reconfigure the backbone network. Secondly, the CBAM attention is added to enhance the feature representation ability of the model, and finally, the loss function Focal EIou and the Smoothed cross-entropy loss function are replaced to improve the detection accuracy, and the improved model is shown in the open dataset B. The model is perfect for open data sets. The enhanced model has an average accuracy increase of 3.5% on the public dataset BDD100K dataset, and the detection rate reaches 41.6 fps in the real-vehicle test, which satisfies both the improvement of the detection accuracy and the requirement of lightweight. Liu Yuqing [7] et al. For the real-time detection of tuna on the mobile side of the YOLOv3 model improvement, first of all, the lightweight backbone network MobileNetv3 to reduce the number of parameters of the model run and the amount of computation, and then replace the SENET module with the CBAM attention module, to further improve the feature extraction ability of the tuna, and finally the use of knowledge distillation to make the tuna detection network more accurate, the results show that the parametric quantity parameter of the detection model is reduced from 234.74 MB to 88.45 MB, the detection accuracy is increased from 93.33% to 95.83%, and the computational speed is increased from 10.12 fps to 15.23 fps. They are making the tuna detection algorithm easier to deploy on mobile devices.

In order to ensure that the model achieves a small increase in detection performance while achieving lightweight deployment, this paper proposes a model lightweight method based on YOLOv8:

1. Since the model’s backbone network uses 3 × 3 convolutional kernels for downsampling and feature extraction, the computational volume is huge and affects the operation speed; this paper replaces the 3 × 3 convolutional kernels used for downsampling the backbone network with the lightweight downsampling convolutional ADown in YOLOv9 [8].
2. To design fast neural networks, it is necessary to satisfy the need to reduce redundant computation and memory access while still extracting spatial features more efficiently, and the proposed PConv in the FasterNet [9] network achieves a much higher operating speed than other networks without affecting the accuracy of various visual tasks. Therefore, this paper uses FasterBlock containing PConv to replace the Bottleneck in C2f and achieve lightness for modelability.
3. The parametric and computational quantities of the detection head account for 40% of the total, and improving the detection head is essential to achieve model lightweight. To this end, the detection head is improved by using shared convolution, and the number of parameters can be significantly reduced, which makes the model lighter, especially in resource-constrained devices. In addition, while using the shared parameters, to cope with the problem of inconsistency in the scale of the target detected by each detector head, the features are scaled using a Scale layer, which allows the detector head to achieve a smaller number of parameters and less computation, and to minimize the loss of accuracy.
4. This paper also uses knowledge distillation to enhance the detection level of the model, which is improved by lightweight.

## 2. Materials and Methods

### 2.1. Data Set Establishment

This data set is selected from the open cone bucket data set FSACOCO [10], with a total of 12,536 photos, which includes all kinds of complex scenes that may appear in the China University Student Driverless Formula Competition, including but not limited to strong light, low light, and diverse road conditions. The dataset was built to provide a rich training platform for universities to cultivate and test their driverless recognition models. The images in the dataset capture key elements on the track—the cone barrels—in a color configuration consistent with the real race scene. The yellow cones mark the start of the track, while the red and blue cones define the inner and outer boundaries of the track, respectively. These cones not only provide visual guidance but also provide key visual cues for the model’s training. In order to ensure the diversity and authenticity of the data, all participating universities use their own cameras for image acquisition and summarize these precious data resources to form a unified and comprehensive data set.

### 2.2. Introduction to the YOLOv8 Network

**Figure 1:**
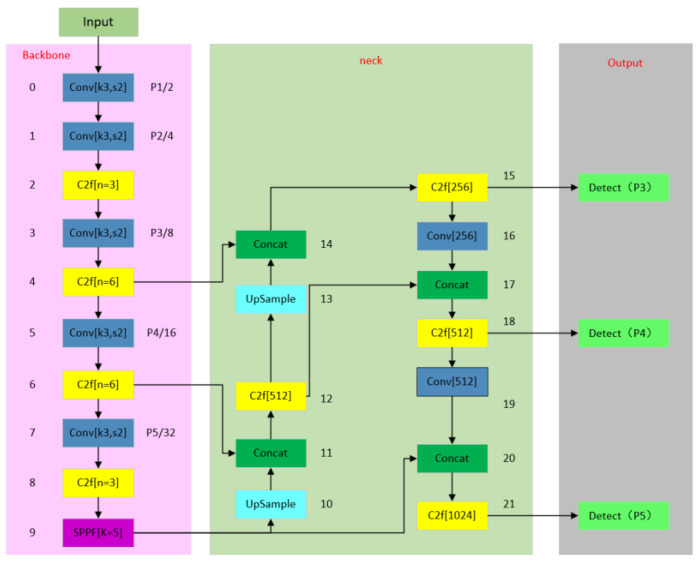
YOLOv8 network structure diagram.

Figure 1 below shows the structure of the YOLOv8 model’s network. The network consists of four main parts: the input, the backbone network, the neck network, and the output.

Input side: Compared with the earlier YOLO algorithm, YOLOv8 adopts the Mosaic data enhancement method on the input side. This method enhances the model’s ability to adapt to variable real-world environments by performing random selection, cropping, and splicing operations on the input images. This approach endows the algorithm with stronger robustness and generalization capabilities and significantly improves the model’s detection performance in complex scenes.

Backbone network: The architecture of YOLOv8 delicately integrates the Conv module, the C2f module, and the SPPF module to form an efficient target detection network: the Conv module is responsible for adjusting the resolution, and the number of channels of the image; the C2f module skillfully integrates the global semantic information and the local target information to enhance the model’s in-depth understanding of the image content; the SPPF module enhances the model’s knowledge of the image content by pooling the features at the same scale by pooling features, enabling the network to perform effective target detection in input images of various sizes.

Neck network: it is mainly used to fuse feature maps from different levels or scales, aiming to improve the model’s ability to process multi-scale scenes. This fusion expands the model’s field of view, making it more adept at handling input images of various sizes.

Output: It introduces a “decoupled head” structure, which separates the extraction of category and location features. YOLOv8 inherits and develops this concept by using two parallel branches to extract category and location features independently and then completes the classification and localization tasks through 1 × 1 convolutional layers, respectively. This design significantly improves the model’s accuracy in localization and adaptability to various types of scenes and enhances the model’s generalizability.

### 2.3. Lightweight Network Design

While pursuing a lightweight model, accuracy maintenance is also crucial. Therefore, the goal of this paper is to deeply optimize the YOLOv8 [11] model without sacrificing detection performance. First, the parameter scale will be compressed, the computational complexity will be reduced, and the model’s memory occupation will be decreased at runtime. Then, after completing the lightweight, the model accuracy will be recovered.

#### 2.3.1. Downsampling Module Replacement

**Figure 2:**
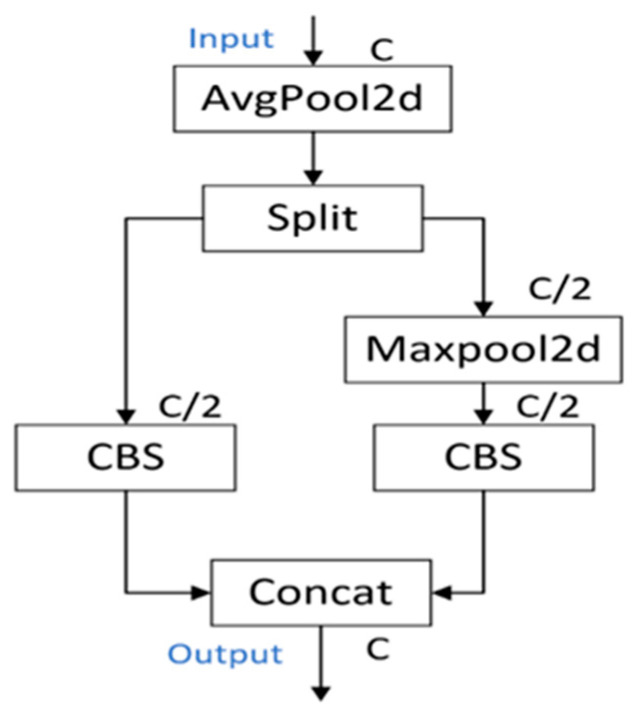
ADown Network Architecture Diagram.

In deep learning models, downsampling is widely used to reduce the spatial dimension of the feature map. Most models use a convolution kernel of 3 × 3 and a step size of 2 to perform downsampling operations. Still, it is very likely that the extraction of important information will be lost by reducing the dimensionality without bias. For this reason, the ADown module in YOLOv9 has redesigned the downsampling module. The specific structure is shown in Figure 2. AvgPool is the average pooling layer, Spilt is mainly used for average distribution, Maxpool is the maximum pooling layer, and the CBS on the left side is a 3 × 3 convolution with step size 2. In contrast, the CBS on the right side adopts a 1 × 1 convolution with step size 1. C denotes the number of input channels, and C/2 denotes that the number of channels becomes half the original.

The ADown module typically consists of the following steps:

1. A convolution operation is performed on the input feature map using a convolution layer to extract features.
2. Downsampling the convolved feature map using a pooling layer to reduce the size of the feature map.
3. Repeat the above steps several times to further reduce the size of the feature map and increase the number of channels.

Main features of the ADown module:

Lightweight design: the ADown module uses a 3 × 3 convolution with 2 steps and a 1 × 1 convolution with 1 step in parallel for downsampling. Compared to the original model that uses 3 × 3 convolution in series for downsampling, the ADown module can reduce the complexity of the model by reducing the number of parameters, which helps to improve the efficiency of the model, especially in resource-constrained environments.

Retention of information: although ADown aims to reduce the spatial resolution of the feature map, its design also focuses on retaining as much image information as possible so that the model can perform target detection more accurately.

Learning ability: The ADown module is designed to have some learning ability, which means it can be adapted to optimize its performance for different data scenarios in the grid.

**Figure 3:**
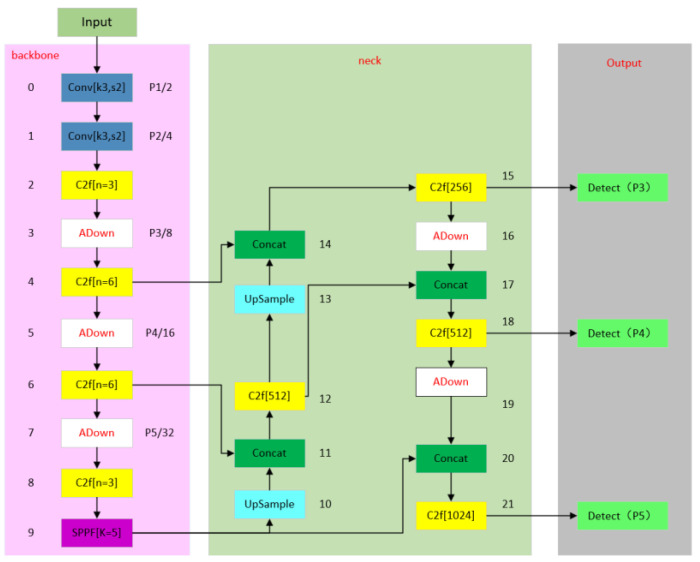
Improved network structure diagram.

Introduced in YOLOv8, it can cut down the number of parameters of the model. The original network structure diagram is shown in Figure 1, and the improved network structure diagram is shown in Figure 3 below.

#### 2.3.2. C2f Module Replacement

**Figure 4:**
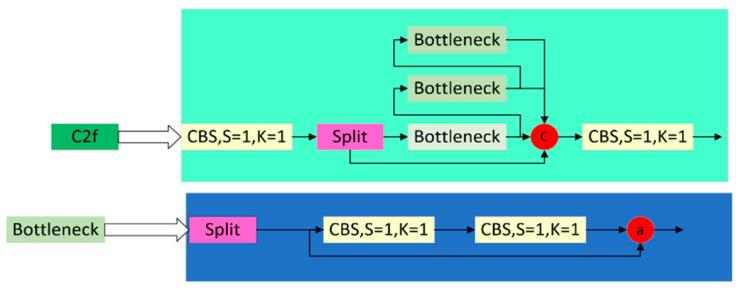
Structure of C2f.

The C2f forward propagation process starts by passing the input data through the first convolutional layer, cv1, and then incorporating the input into two parts. One part is directly forwarded to the output, and multiple Bottleneck modules process the other part. Finally, the results of the two parts are spliced in the channel dimension and passed through the second convolutional layer, cv2, to get the final output. The structure is shown in Figure 4 below.

Role of C2f module:

Feature Transformation: C2f performs feature transformation on the input data employing two convolutional layers (cv1 and cv2). The cv1 convolutional layer transforms the number of channels of the input data from c1 to 2 * self.c, and the cv2 convolutional layer transforms the number of channels of the feature map after a series of operations from (2 + n) * self.c to c2. These convolutional operations help extract features from the input data at different levels and provide abstraction levels of features in the input data.

Branching: The C2f module divides the input data into two branches for processing. One branch is directly passed to the output, and the other branch is processed by multiple Bottleneck modules. Such a branching design helps increase the network’s nonlinear and symbolic capabilities, thus improving its ability to model complex data.

Feature Fusion: the C2f module achieves feature fusion by splicing features from different branches in the channel dimension. The spliced features will contain information from various branches, enriching the feature representation capability.

**Figure 5:**
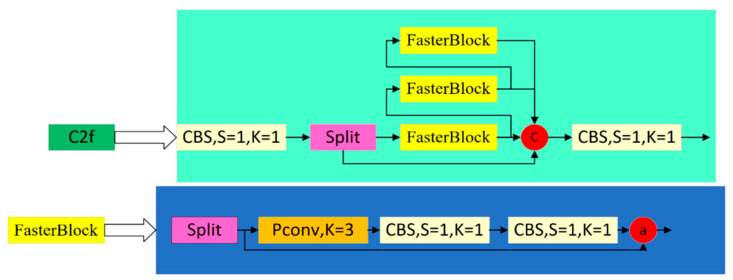
Structure of C2f_Faster.

This paper adopts the FasterBlock module in the FasterNet network to replace the Bottleneck in C2f and design a lighter and faster detection network. The structure of the replaced C2f_Faster module is shown in Figure 5 below.

The most significant difference between the C2f_Faster module and the C2f_Faster module is the use of Pconv (Partial Convolution), whose core idea lies in its flexibility and adaptability to missing data. Compared to traditional convolution operations, partial convolution does not mechanically apply the same convolution kernel to all parts of the input data. Instead, it dynamically determines the convolution kernel’s scope based on the data’s validity, i.e., whether the data points are missing or corrupted. The FasterBlock module, which is based on Pconv, allows for more efficient extraction of spatial features by reducing the number of redundant computations and memory accesses. This makes the detection model more lightweight.

#### 2.3.3. Detection Head Replacement

**Figure 6:**
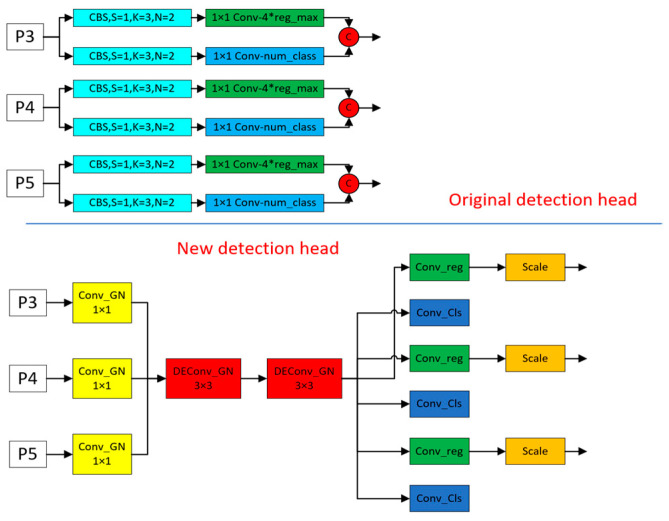
Diagram of the structure of the old and new inspection heads.

The improvement in the head is indispensable to realize a lightweight detecting model. In this work, the authors created a new head called LSDECD since the computation of the model occupied 40% of its parameters in the head. This was meant to make the detection model in order to continue improving the accuracy of detection. The structure of the new detection head is shown in Figure 6 below.

**Figure 7:**
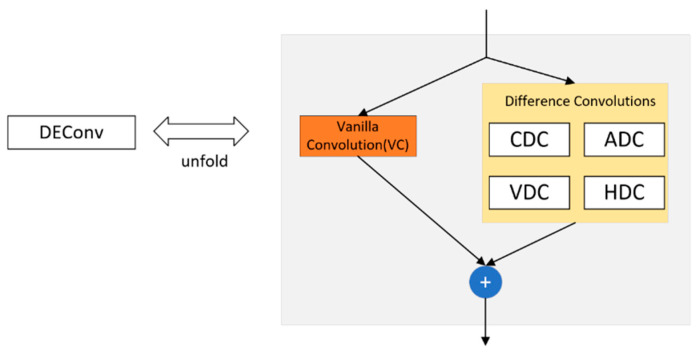
Structure of DEConv.

Characteristics of the new detection header:

1. Replace all the normalization layers in the convolution with Group Normalization (GN) [12]. Group Normalization is proposed to solve the problem that the model is microscopic because of the batch_size (each training model is trained by selecting a batch_size of samples in the training dataset) in the training process, which leads to the model With the rapid development of deep learning, the models we use are getting bigger and bigger, which also leads to the problem that a batch occupies more and more video memory during training, resulting in a small batch_size on a video card, which ultimately leads to a significant deterioration of the model effect. Group Normalization has been demonstrated to improve the detection performance in the FCOS [13] paper. Group Normalization is also shown in the FCOS paper to improve the localization and classification performance of the detection head.
2. The problem of parameter count and computation proliferation in the detection head mainly lies in the fact that when it performs classification and localization, the detection head carries out feature extraction three times, which is not conducive to deploying the model on small devices. Therefore, to make the detection head lightweight, the design of the parameter sharing is to put the three-time feature extraction in one piece and then use the scale module to scale the scale.
3. The convolution for feature extraction is replaced with DEConv (detail-enhanced convolution) [14]. The structure is schematically shown in Figure 7 as follows.

DEConv contains five convolutional layers (four difference convolution kernels and one regular convolution) that are deployed in parallel for feature extraction. Specifically, central difference convolution (CDC), angular difference convolution (ADC), horizontal difference convolution (HDC), and vertical difference convolution (VDC) are used to integrate traditional local descriptors into the convolutional layers, which allows for enhanced representation and generalization capabilities. In difference convolution, pixel differences in an image are first computed and then convolved with a convolution kernel to generate an output feature map, and by designing a difference computation strategy for pixel pairs, a priori information can be displayed and encoded into the CNN. These convolutions are used for feature extraction and learning, enhancing representation and generalization capabilities.

**Figure 8:**
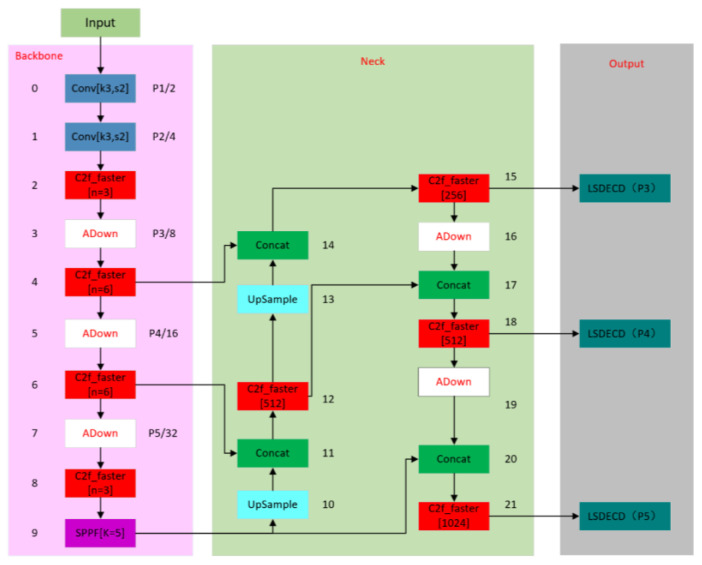
Structural diagram of the final network.

The final network structure diagram summarizing the three lightweight methods is shown in Figure 8 below.

Finally, the main improvement of the network is divided into three parts. The first part is improved by using the red module, replacing the original C2f module with C2f_faster, which has the advantage of a lightweight detection network. The second part is improved by using the white module, replacing the original extraction module with the ADown module. The advantages of this module can not only improve the extraction efficiency but also reduce the number of model parameters and the amount of calculation. In the third part, the dark green module is used to improve the display, and the original detection head is changed to the self-developed LSDECD detection head. The advantages of this module are that it not only improves detection efficiency but also helps the lightweight model.

### 2.4. Knowledge Distillation

To recover the problem of decreasing the accuracy of detection models in lightweight processing, this paper adopts the method of knowledge distillation. Knowledge distillation is to extract the knowledge of a parametric, computationally large, and high detection level model and pass it to a small student model, which can be understood as a large teacher neural network teaching its knowledge to a small student network here, there is a knowledge migration process from the teacher network to the student network, the teacher network is generally bloated, so the teacher network teaches the knowledge to the student network, the student network is smaller so that the student network can be used to do things that lighter networks do [15].

**Figure 9:**
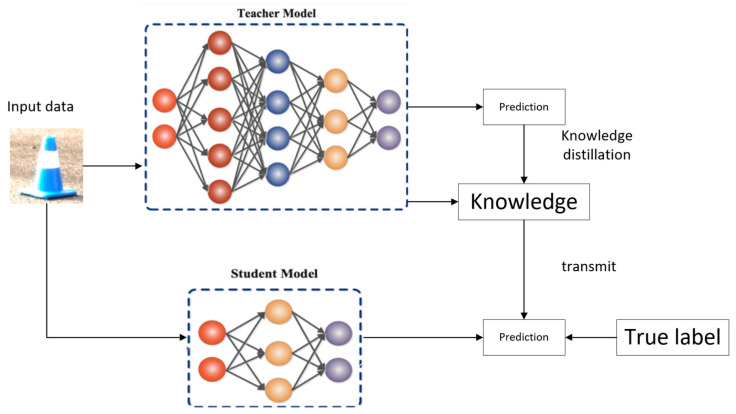
Knowledge Distillation Diagram.

Knowledge distillation uses the Teacher-student model, in which the Teacher is the “knowledge” exporter and the Student is the “knowledge receiver”; the process of knowledge distillation is divided into two stages:

1. Original model training: training the “Teacher model”, referred to as Net-T, which is characterized by a relatively complex model and can also be integrated from multiple separately trained models. The teacher model is not restricted, but the only requirement is that input X can output Y, where Y is mapped by softmax, and the output value corresponds to the probability value of the corresponding category.
2. Streamlined model training: train the student model, or Net-S, a single model with a small number of parameters and a relatively simple model structure. Similarly, input X can output Y, which can also output the probability value corresponding to the corresponding category after softmax mapping.
3. The teacher model has a strong learning ability and can transfer what it has learned to the student model, which has a relatively weak learning ability, to enhance its generalization ability. Finally, the student model is deployed online for the prediction task. The knowledge distillation schematic is shown in Figure 9.

Characteristics of knowledge distillation:

1. Improve model accuracy: If you are not very satisfied with the accuracy of the current network model A, you can train a model B with high accuracy (usually with a more significant number of parameters and a larger time delay). Then, you can use model B as a teacher model to distill the knowledge of the student model A so that you will get a model with higher accuracy after training.
2. Reduce the model delay and compress the network parameters: If you are not satisfied with the delay of the current network model A, you can find a model B with low delay and smaller number of parameters, which usually has relatively low precision, and then train a higher precision teacher model C to distill the knowledge of this model B with small number of parameters, to make the precision of this model B close to the most original model A, to achieve the goal of reducing the time delay. Model A, thus fulfilling the purpose of lowering the timeliness.
3. Domain migration between labels: If we use the dog and cat dataset to train a teacher model A and the watermelon and strawberry dataset to train a teacher model B, then we should be able to use these two models to distill a model that can recognize dogs, cats, watermelons, and strawberries at the same time so that we can integrate and migrate the datasets of two different domains.

Classification of knowledge distillation: Knowledge distillation is the ability to migrate the model according to the different migration methods. It can be simply divided into based on logic (Logits) distillation, based on features (Feature) distillation, and a combination of the two distillation algorithms.

Logic distillation: the common point of classification problems is that the model will have a softmax layer at the end, and its output value corresponds to the probability value of the corresponding category. In knowledge distillation, since we already have a Teacher model with solid generalization ability, we can directly let the Student model learn the generalization ability of the Teacher model when we use the Teacher model to distill and train the Student model.

Feature distillation: Learning the features of the middle layer in the Teacher network structure forces the Student to approximate the network response of the corresponding middle layer of the Teacher to a particular middle layer. In this case, the reactions of the Teacher’s intermediate feature layers are the knowledge passed to the Student, which is essentially the feature-level knowledge that the Teacher migrates to the Student [16].

**Figure 10:**
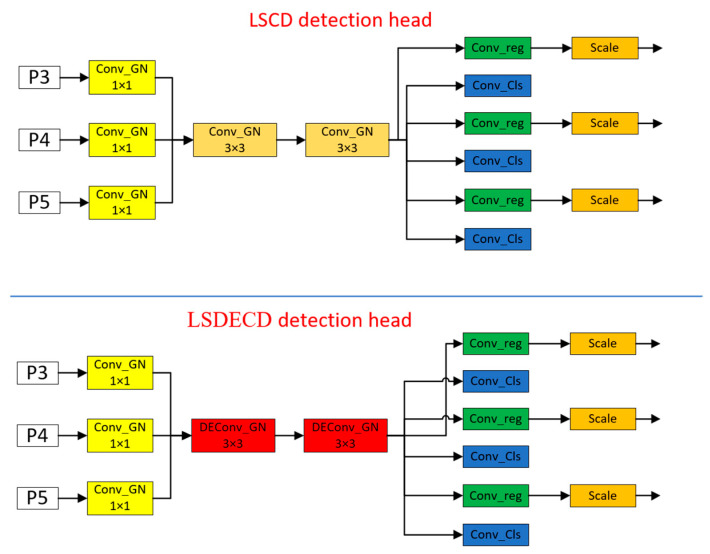
Schematic structure of LSCD detection head.

The structure of the self-research detection head LSCD is shown in Figure 10 below, which is characterized by a large number of parameters but high accuracy compared to the LSDECD detection head, which has roughly the same structure but the convolution that performs the feature extraction uses a 3 × 3 convolution kernel normalized to Group Normalization.

In this paper, we choose the previously described public dataset FSACOCO dataset as the most detection input for the knowledge distillation experiment, the model processed by the lightweight method as the most small-model student network, and YOLOv8x plus the self-developed detection head LSCD as the most large-model teacher network. Using a combination of feature and logistic distillation methods, we transfer the relevant information from the teacher network to the student network so that it has a high detection level. The same training strategy as the model training is used in the training process, and the number of training rounds is set to 200. The reason for choosing 200 rounds is that it is found in the training that the model tends to converge after 150 rounds. If the number of training rounds is increased, again and again, the calculation will be more complicated, so this paper chooses a more reasonable 200 rounds.

## 3. Experiments and Analysis of Results

### 3.1. Test Environment and Evaluation Indicators

#### 3.1.1. Test Environment Configuration

The experiments were conducted on a Linux operating system with an RTX 4090 GPU, 12th Gen Intel Core i5-12400, and 24 GB RAM utilizing the Pytorch 1.13.0 deep learning framework, CUDA 11.7, and a Python 3.8 software environment. The real vehicle used for the validation session was a racing car used in the Chinese university driverless formula competition. 3.8. For the real car verification session, the real car used is the one used in the Formula Unmanned Driverless Competition for Chinese university students.

#### 3.1.2. Evaluation Index of the Test

To show the impact of lightweight processing on the model, this paper selected 2 model recognition performance indicators, two computational performance indicators, and model memory usage to evaluate the model. Model recognition performance includes Recall (*R*) and mean average precision (*mAP*) with an IOU threshold of 0.5, and calculation performance indicators include parameter number and floating point calculation amount.

The calculation formula of Recall (*R*) is shown in Equation (1). It represents the proportion of positive samples correctly identified by the model in all positive samples. The higher the recall rate, the less the model misses the target detection [17].

(1) $$R=\frac{TP}{TP+FN}$$

where *TP* represents the number of samples correctly predicted, *FN* represents the number of samples incorrectly predicted as negative, and *FP* represents the number of samples incorrectly predicted.

The calculation formula of average accuracy (*mAP* 0.5) with an IOU threshold of 0.5 is shown in Equation (4). The larger the value of *mAP*, the higher the detection accuracy of the detection model.

(2) $$P=\frac{TP}{TP+FP}$$

(3) $$AP=\int_{0}^{1}P(R)$$

(4) $$mAP=\frac{\sum_{i=1}^{N_{Class}}AP_{i}}{N_{class}}$$

The parameter represents the complexity of the model. The larger the parameter is, the more computational resources the model occupies, and the computational amount represents the computational cost used in the operation of the model [18].

#### 3.1.3. Distillation Test

This paper’s distillation combines logic and characteristic distillation, and the teacher model adopts the YOLOv8x model of the LSCD detection head. The test results are shown in Table 1 below.

It can be seen from the table that in terms of accuracy, the use of detection head LSCD is superior to the use of detection head LSDECD in the teacher model, and in terms of lightweight, LSDECD is superior to LSCD. Since the main focus of the teacher model is to achieve high-precision detection, this paper chooses the combination of YOLOv8x and LSCD as the teacher model. Knowledge is transferred to the YOLOv8n network after lightweight means so that the distillation model ensures that it maintains high accuracy and significantly improves its practicality and flexibility in resource-constrained environments.

#### 3.1.4. Ablation Test

To further verify the effectiveness of different improved methods proposed in this paper for improving the detection model on the racing cone bucket, the enhanced algorithm’s ablation test was conducted on the public data set FSACOCO, and the test results are shown in Table 2.

In the table, YOLOv8n-All represents the result of improving the model by combining all the lightweight methods. As seen from the table, each step of the lightweight method can reduce the number of parameters and calculations, and each part is indispensable. The LSDECD detection head can achieve lightweight and improve accuracy. Finally, knowledge distillation can ensure the original lightweight results and further enhance the detection accuracy of the model. It greatly meets the characteristics of high detection accuracy in small model structures and satisfies all the requirements for deployment of tiny devices in the race car of the China University Student Driverless Formula Competition.

#### 3.1.5. Contrast Test

To prove the effectiveness of the proposed method, the improved method was compared with YOLOv5 [19], YOLOv6 [20], YOLOv7 [21], YOLOv7-Tiny, RT-DERT [22], and RT-DERT-R50 in the table, and the results are shown in Table 3.

It can be clearly seen from the results table that the model proposed in this paper outperforms other detection algorithms in four key performance indicators, namely recall rate, average accuracy (*mAP*), parameter number, and computation amount. Although the detection accuracy is slightly lower than that of the YOLOv7 algorithm, our model exceeds it in terms of the number of model parameters and the amount of model computation. The number of parameters and the amount of computation determine the model’s computing and detection speeds. This paper mainly studies the deployment of small devices. Our improved algorithm is more suitable.

### 3.2. Preparation for Real Vehicle Verification

In order to thoroughly verify the practicality and effectiveness of the algorithm, the algorithm is deployed on the actual vehicle for verification. Before officially using the camera on the vehicle for data acquisition, the camera is first calibrated with accurate internal parameters. This step is crucial because it ensures the accuracy and reliability of the image data captured by the camera. The camera lens distortion can avoid the adverse effect on the target detection result. The camera used in the experiment is the Mercury II industrial camera MER2-231-41U3C as the camera sensor, and real-time image data is sent to the algorithm through the ROS operating system for verification.

**Figure 11:**
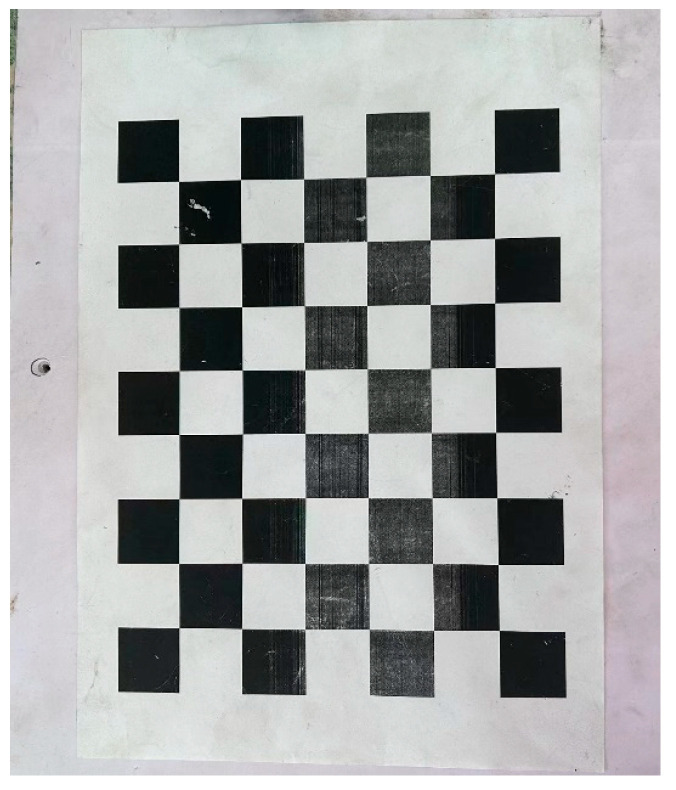
Internal reference calibration board.

The Zhang Zhengyou [6] camera calibration method was adopted to calibrate the camera’s internal parameters. The international standard chess black-and-white grid calibration board was selected, and it is shown in Figure 11 below. The side length of each square was 25 cm, the length of the corner was set to 7, and the width was set to 6.

**Figure 12:**
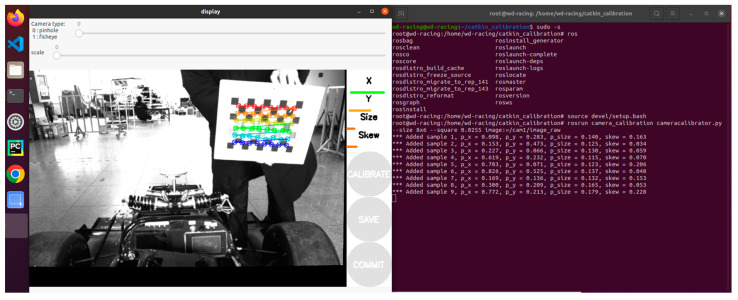
Camera calibration interface.

Calibration process: First, open the camera calibration function package, then enter the calibration interface, and slowly move the calibration board in front of the camera from front to back, from left to right, and from top to bottom until the progress bar under X, Y, Size, and Skew on the right side of the program interface turns green, the calibration is completed. Finally, click SAVE to save the calibration result, and put the final parameter matrix into the camera driver’s configuration file to complete the parameter replacement. The camera calibration interface is shown in Figure 12 below.

### 3.3. Analysis of Real Vehicle Results

In this paper, two scenarios, the typical scene and the light entity scene are selected to verify the feasibility of the improved algorithm in the two scenarios under the same scene and the same vehicle behavior.

**Figure 13:**
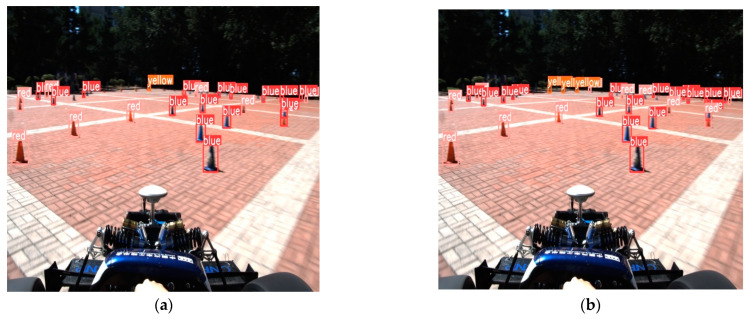
Detection results in normal scenarios: (**a**) The original YOLOv8; (**b**) The improved YOLOv8.

The first is accurate vehicle detection under everyday scenarios, as shown in Figure 13 below.

**Figure 14:**
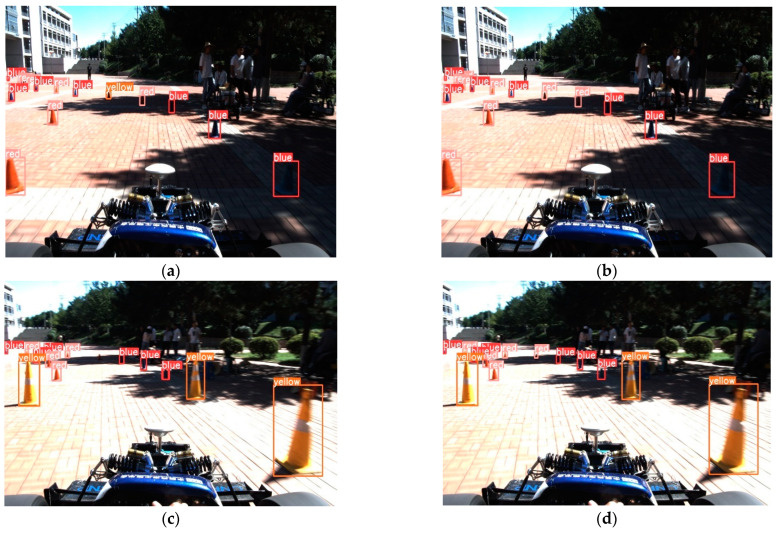
Detection results under strong light (**a**) The original YOLOv8; (**b**) The improved YOLOv8; (**c**) The original YOLOv8; (**d**) The improved YOLOv8.

The second is the case of stronger light detection, as shown in Figure 14 below.

The comparison of the two scenarios shows that in the ordinary environment, the improved model achieves lightweight and improves the detection effect. Under the light solid condition, through the comparative analysis of Figure 14a,b, we find that the improved algorithm can effectively reduce the false positives. In comparing Figure 14c,d, the enhanced algorithm also effectively reduces the phenomenon of underreporting.

Based on the above analysis, the improved algorithm improves the detection level, reduces false detection and leakage, and reduces memory consumption by optimizing the model structure and meeting the deployment needs on small devices.

## 4. Conclusions

The central goal of this thesis is to deploy a conical bucket detection model on small, resource-constrained devices. The main challenge we face in this process is how to improve the detection accuracy while ensuring that the model is lightweight so that the model can run effectively on these devices. Based on this goal, we have investigated the YOLOv8 algorithm and successfully developed a novel detection model. The model not only meets the deployment requirements on small devices but also has high detection accuracy. It is mainly reflected in the following aspects:

In terms of lightweight: First, the convolutional module for downsampling in the network is replaced, and the ADown module is used for downsampling. This method not only realizes that it is lightweight but also optimizes the network and improves the recall rate of the detection model. Secondly, in order to compress the model further, the bottleneck module of C2f in the YOLOv8 network is replaced by FasterBlock in the FasterNet network, which implements network compression. Finally, considering the bottleneck of the number of detection headers and a large number of computing resources, the bottleneck module of C2f is replaced with Fasterblock in the Faster network. Improve the detection head. Replace the original detection head with the LSDECD detection head. Light weight is achieved, and the detection accuracy is improved.

In terms of improving accuracy, Since the detection model processed by a series of lightweight means has a low detection level, knowledge distillation is adopted to take the YOLOv8x + LSCD detection model as the teacher model and the detection model processed by a series of lightweight means as the student model. The method of combining logical distillation and characteristic distillation is used to transfer knowledge to the student model. The algorithm proposed in this paper can make the detection model more effective in detecting obstacles and improve the detection efficiency.

Finally, the improved model’s accuracy, recall rate, and average accuracy on the FSACOCO dataset are 92.7%, 84.6%, and 91%, respectively. Compared with the original YOLOv8n detection model, the recall rate and average accuracy increased by 2.7 and 1.2 percentage points, the memory was half of the original, and the model calculation amount was 53%. The model significantly reduced the cone bucket missing detection phenomenon in the actual vehicle test. At the same time, the detection speed is ensured to meet the deployment requirements on small devices.

Compared to the literature [23], the improved method proposed in this thesis has a significant advantage in terms of model weight, although it is slightly inferior in terms of detection accuracy. The model in this study outperforms this literature in terms of the number of parameters, computation, and memory footprint, which makes the model easier to deploy on small resource-constrained devices. In addition, this study utilizes a publicly available dataset for model training, whereas this literature relies on constructing datasets from photographs acquired through cameras, which may result in the effect of lighting variations on the detection effect being ignored in some specific scenarios, such as lighting variations. Therefore, the method in this study has wider applicability and flexibility in practical applications.

Compared with the literature [2], which mainly focuses on the improvement of detection accuracy and thus ignores the size and detection efficiency of the detection model, this paper fully considers the detection efficiency and improves the detection speed and memory of the detection model while improving the detection accuracy as much as possible. This paper also has the disadvantage of not using public datasets.

Although the focus of this paper is on lightweight, the work to improve the detection accuracy remains to be completed. It is hoped that alternative methods can be used so that their detection models can fulfill both lightweight and high-accuracy requirements. The improved method in this paper can be applied to cone-bucket detection in complex scenarios, and the improved idea can be carried over to the detection of other small targets.

## Figures and Tables

**Table 1:** Knowledge distillation test results.

Model	*P*/%	*R*/%	*mAP*/%	Parameter Quantity	Floating-Point Computation	Model Memory/MB
YOLOv8x-LSDECD	93.6	88.4	94.2	61.7 × 10^6^	250.3 × 10^9^	118
YOLOv8x-LSCD	94.6	87.9	94.3	68.1 × 10^6^	250.3 × 10^9^	128
YOLOv8x	92.9	85.1	92.5	68.1 × 10^6^	257.4 × 10^9^	130

**Table 2:** Ablation test results.

Model	*R*/%	*mAP*/%	Parameter Quantity	Floating-Point Computation	Model Memory/MB
YOLOv8n	81.9	89.8	3.0 × 10^6^	8.1 × 10^9^	6
YOLOv8n-ADown	82.6	89.7	2.59 × 10^6^	7.4 × 10^9^	5
YOLOv8n-C2f_Faster	81.7	88.8	2.30 × 10^6^	6.3 × 10^9^	5
YOLOv8n-LSDECD	85.1	91.5	2.36 × 10^6^	6.5 × 10^9^	5
YOLOv8n-All	83.5	90.1	1.37 × 10^6^	4.3 × 10^9^	3
YOLOv8n-All-distill	84.6	91	1.37 × 10^6^	4.3 × 10^9^	3

**Table 3:** Comparative test result.

Model	*R*/%	*mAP*/%	Parameter Quantity	Floating-Point Computation
YOLOv5s	84	90.8	9.11 × 10^6^	23.8 × 10^9^
YOLOv6s	82.8	89.9	16.2 × 10^6^	44 × 10^9^
YOLOv7	91	95.5	29.1 × 10^6^	103.2 × 10^9^
YOLOv8n	81.9	89.8	3.0 × 10^6^	8.1 × 10^9^
RT-DERT-x	78.8	86.6	28.4 × 10^6^	100.6 × 10^9^
RT-DERT-r50	83.7	90.3	41.9 × 10^6^	125.6 × 10^9^
Ours	84.6	91	1.37 × 10^6^	4.3 × 10^9^

## Data Availability

The data used to support the findings of this study are available from the corresponding author upon request.
